# Housekeeping gene expression stability in reproductive tissues after mitogen stimulation

**DOI:** 10.1186/1756-0500-6-285

**Published:** 2013-07-22

**Authors:** Marcia Arenas-Hernandez, Rodrigo Vega-Sanchez

**Affiliations:** 1 vDepartment of Nutrition Research, Instituto Nacional de Perinatología Isidro Espinosa de los Reyes, Montes Urales 800, Lomas Virreyes, Mexico City, Mexico

**Keywords:** Expression stability, Reproductive tissues, Housekeeping genes, Lipopolysaccharide, Phytohemagglutinin

## Abstract

**Background:**

Intrauterine infection during pregnancy can trigger a local inflammatory response leading to several complications, such as preterm labor. Many studies have used *in vitro* and *in vivo* models employing mitogens to induce the expression of the characteristic proinflammatory mediators triggered by infection. However, relative expression assays depend on the stability of housekeeping gene expression, which can vary depending on certain stimuli. In this study, we analyzed the stability and pairwise variation in the expression of *GAPDH*, *ACTB* and *RNA18S1* in cultured reproductive tissues under mitogen stimulation. We used fetal membranes, placental villous and umbilical cord explants from patients with normal term pregnancies (>37 weeks of gestation), as well as myometrium and cervix explants from patients undergoing hysterectomies. Tissues were stimulated with lipopolysaccharide or phytohemagglutinin for 24 hours. We then analyzed the expression stability and the pairwise variation of *GAPDH, ACTB* and *RNA18S1* from real time quantitative RT-PCR absolute threshold cycles (Cp) using geNorm software.

**Results:**

In all of the tissues, the three housekeeping genes showed great stability under our experimental conditions. Pairwise variation analyses showed that only two reference genes are required for adequate normalization, *GAPDH* and *ACTB* being optimal in the cervix, fetal membranes and umbilical cord, while *GAPDH* and *RNA18S1* are best for normalization in the placenta and myometrium.

**Conclusion:**

Our results show that *GAPDH*, *ACTB* and *RNA18S1* are adequate references for gene expression normalization in reproductive tissues stimulated with mitogens in culture.

## Background

The molecular mechanisms underlying pregnancy complications such as ascending intrauterine infection have been explored using both *in vivo* and *in vitro* models in which mitogens are commonly used to emulate the characteristic proinflammatory response triggered by such an infection [1-3].

In these models, gene expression of effector markers, such as inflammatory mediators, prostaglandins, matrix metalloproteinases, etc., is commonly examined. Relative expression analyses, however, require that one or more housekeeping genes be used as references for normalizing the expression of target genes. Some of the most commonly used housekeeping genes are those encoding for glyceraldehyde 3-phosphate dehydrogenase (*GAPDH*), beta-actin (*ACTB*) and the 18S fraction of ribosomal RNA (*RNA18S1*).

The accountability and reproducibility of relative expression analyses depends greatly on whether the expression of these genes remains stable (i.e. constitutive) regardless of the cell type, tissue or the particular experimental conditions.

However, several studies have shown that housekeeping gene expression can vary under certain stimuli [4-7], making it essential to analyze their stability in each of the particular experimental conditions.

Despite these evidences, the stability of housekeeping genes has been scarcely explored in human reproductive tissues, namely within the placenta and to an even lesser extent within the cervix and myometrium [8-13]. Moreover, the few available reports provide contradictory results; for example, Meller et al., Cleal et al., and LifeTechnologies found that *GAPDH* was too varied within the placenta and was therefore unreliable as a reference, yet Murthi et al. found it to be stable and suitable enough for normalization [8,10,11,13]. Housekeeping gene stability, however, has not been studied in these tissues- neither in culture, nor under stimulation conditions.

In this study, we aimed to analyze the stability and pairwise variation in the expression of *GAPDH*, *ACTB* and *RNA18S1* within cultured human myometrium, cervix, villous placenta, fetal membranes and umbilical cord after being stimulated with two mitogens, lipopolysaccharide (LPS) and phytohemagglutinin (PHA).

## Methods

### Patients and biological samples

This project was approved by the Internal Review Board of the Instituto Nacional de Perinatologia Isidro Espinosa de los Reyes in Mexico City (Registration number 212250–02191) and informed consent was obtained from all participating women.

Fetal membranes (n=3), placental villous explants (n=3) and umbilical cords (n=3) were obtained immediately after delivery from the placentae of patients with normal term pregnancies (>37 weeks of gestation), regardless of delivery mode (vaginal or cesarean section) or the presence of active labor.

Myometrium (n=5) and cervix (n=3) samples were also obtained from patients undergoing hysterectomies due to uterine leiomyomas. However, all of the tissue sections used in this study exhibited a normal histology and coloration with no apparent signs of hypertrophy or necrosis.

Subsequently, all tissues were transported and then washed in sterile saline to remove blood and debris.

### Tissue culture and stimulation

Tissue explants of myometrium, cervix, villous placenta and umbilical cord, approximately 1 cm^3^ in measurement, as well as 1 cm^2^ explants of fetal membranes were cultured for 24 h in DMEM supplemented with 1% lactoalbumin hydrolysate, 1% sodium pyruvate and 1% antibiotic-antimycotic (penicillin G sodium, streptomycin sulfate, amphotericin B), all reagents from Gibco BRL (Grand Island, NY, USA). Tissues were cultured in 12 well, flat bottom culture plates, with low evaporation lids (BD Falcon, Franklin Lakes, NJ, USA).

Viability of all tissues was confirmed throughout the culture using the Cell Proliferation Kit II (XTT) according to the manufacturer’s protocol (Roche Applied Science, Mannheim, Germany).

Tissues were stimulated with 1 μg/ml of LPS from *Escherichia coli* (055:B5) or 10 μg/ml of PHA from *Phaseolus vulgaris* (Sigma Chemical, St. Louis, MO, USA). Both stimulated tissues and unstimulated controls were cultured in duplicate.

After culture, tissues were immediately placed in 1 ml of Trizol (Invitrogen, Carlsbad, CA, USA) and stored in ice for 30 min to allow the reagent to penetrate the tissue. Samples were then stored at −20°C until further processing.

### RNA isolation and cDNA synthesis

Tissues in Trizol were thoroughly homogenized with sterile scissors and then centrifuged at 12,000 × g for 10 min at 4°C in order to remove insoluble material. The homogenate was recovered and the total RNA was isolated according to the manufacturer’s protocol. Approximately 3 μg of each RNA sample were used to synthesize the complementary DNA (cDNA) with the Transcriptor First Strand cDNA Synthesis Kit (Roche Applied Science, Mannheim, Germany), using random hexamer primers. The reverse transcription reaction was carried out in a Mastercycler Gradient equipment (Eppendorf, Hamburg, Germany) at 25°C – 10 min / 55°C – 30 min / 85°C – 5 min. The synthesized cDNA was stored at −20°C until it was later used.

### Real-time PCR

Quantitative real-time PCR was performed in a Light Cycler 480 instrument using a Light Cycler 480 Probes Master kit and TaqMan Probes (hydrolysis probes labeled with fluorescein) according to the manufacturer’s protocol (Roche Applied Science, Mannheim, Germany). Specific primers for mRNA sequences of *GAPDH* (forward 5’-AGCCACATCGCTCAGACAC-3’; reverse 5’-GCCCAATACGACCAAATCC-3’), *ACTB* (forward 5’-ATTGGCAATGAGCGGTTC-3’; reverse 5’-GGATGCCACAGGACTCCAT-3’) and *RNA18S1* (forward 5’-CGATTGGATGGTTTAGTGAGG-3’; reverse 5’-AGTTCGACCGTCTTCTCAGC-3’) were designed using the ProbeFinder software accessible at http://www.universalprobelibrary.com. TaqMan probes #60, 11 and 81 were used respectively.

The primers for *GAPDH, ACTB* and *TNF* were designed to have intron spanning sequences, so as to avoid false positive signals from possible residual genomic DNA. Twenty nanograms of sample cDNA were added to each reaction. Real-time PCR conditions were as follows: one cycle at 95°C, 5 min; 55 cycles of denaturation (95°C, 10 sec), annealing (60°C, 20 sec) and extension (72°C, 1 sec).

*TNF* expression (forward 5’-CAGCCTCTTCTCCTTCCTGAT-3’; reverse 5’-GCCAGAGGGCTGATTAGAGA-3’; probe #29) was also analyzed as an experimental control to ensure that tissues were responsive to mitogen stimulation [14]. Fold changes in *TNF* relative expression were calculated using the 2^-ΔCt^ method, using each of the three housekeeping genes as references.

### Analysis of gene stability and variability

The geNorm VBA applet for Microsoft Excel (available at http://medgen.ugent.be/genorm/) was used to analyze gene stability and variability, in accordance with the official software instruction. This application calculates the gene expression stability measure for a reference gene (*M*), as the average pairwise variation for that gene, against all other tested reference genes. Stepwise exclusion of the genes with the highest *M* value allows for ranking the tested genes according to their expression stability. Genes with the smallest *M* value (lower than 1.5) are considered the most stable.

Pairwise variation (Vn/n+1) was also calculated in order to evaluate the minimum number of genes required for normalization in comparative gene expression analyses. According to Vandesompele et al., a pairwise variation of 0.15 was considered the cut-off value below which the inclusion of an additional reference gene would not be required [15].

Since geNorm requires normalized raw expression levels (not Ct values), we used Light Cycler 480 SW 1.5 software (Roche Applied Science, Mannheim, Germany) to obtain absolute threshold cycles (Cp) of *GAPDH*, *ACTB* and *RN18S1* of each sample. All samples showed Cp values < 40.

### Statistical analysis

Differences in viability of gestational tissues between basal time and 24 h in culture were evaluated with the Wilcoxon test in SPSS v.17 software. Differences with P ≤ 0.05 were considered significant.

## Results

**Figure 1:**
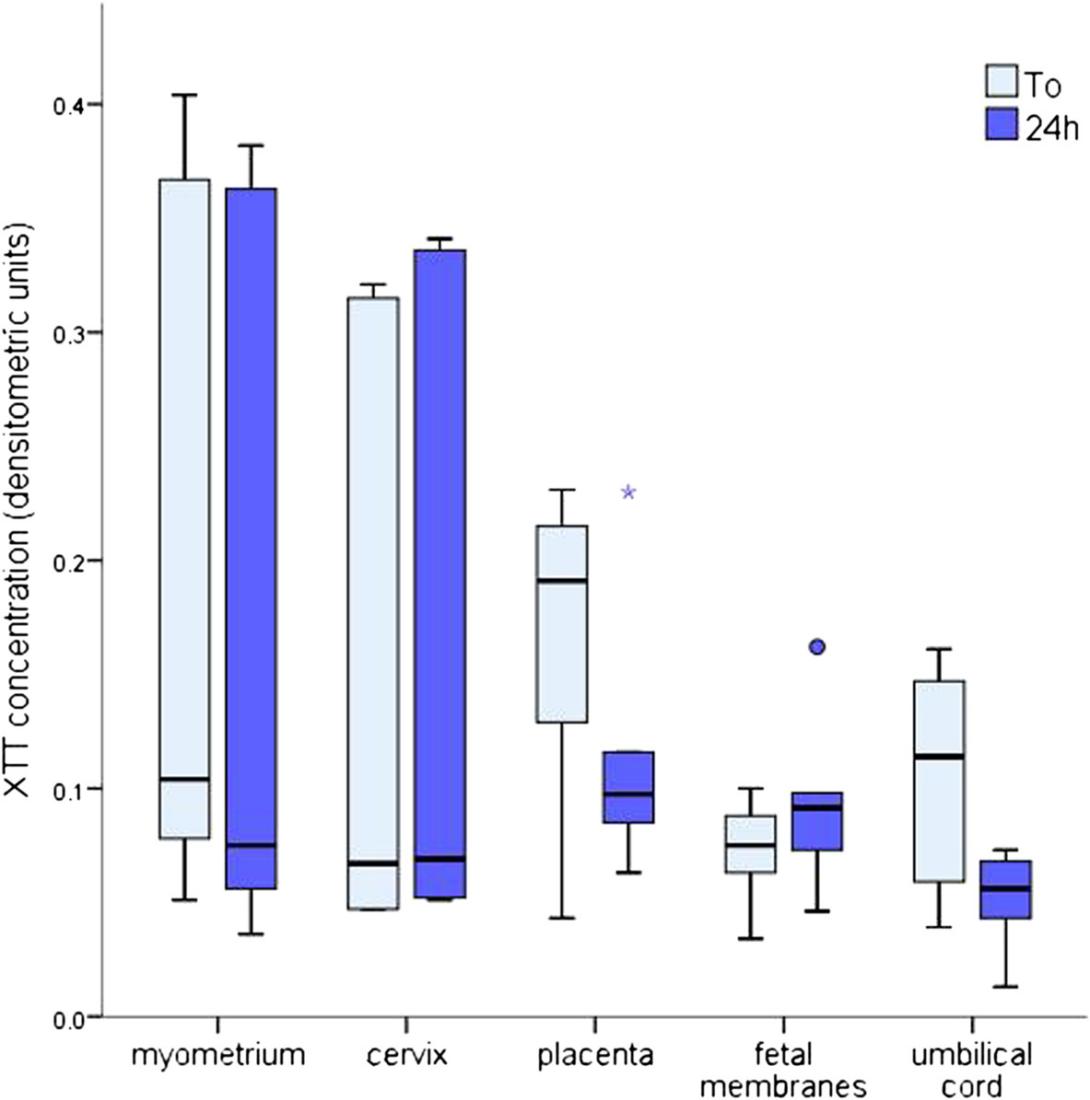
**Viability of reproductive tissues during culture.** The values represent median densitometric units with interquartile ranges. Outlier values are represented by circles and asterisks. No differences were found in the viability of any tissue comparing 0 h and 24 h with the Wilcoxon test (P > 0.05).

There was no significant difference in the viability of any tissue throughout the 24 h culture period (Figure 1).

**Figure 2:**
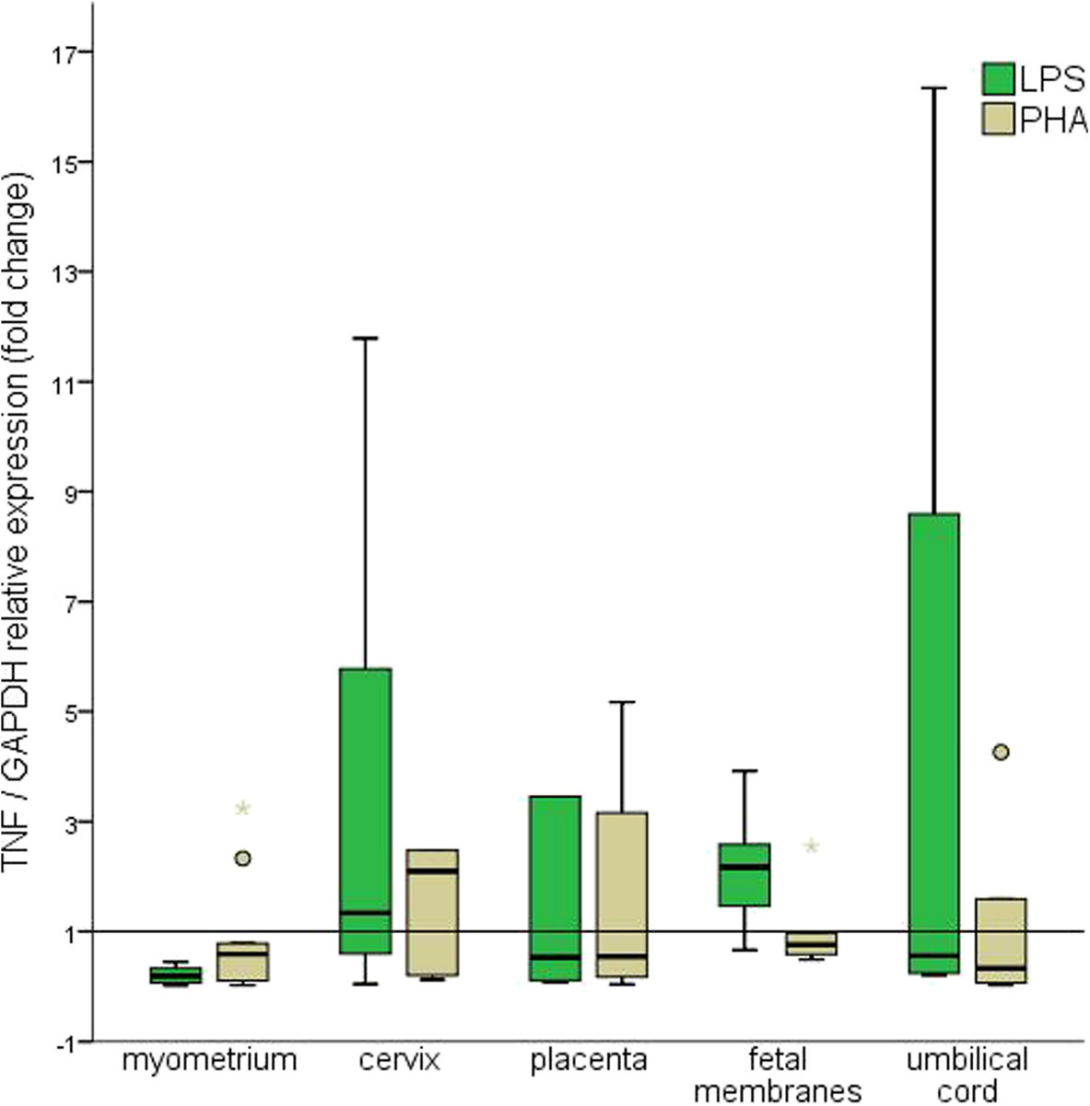
**Fold changes in *TNF *relative expression using *GAPDH *as reference.** The 2^-ΔCt^ method was used to calculate fold changes. The values represent median fold change with interquartile ranges; Outlier values are represented by circles and asterisks.

**Figure 3:**
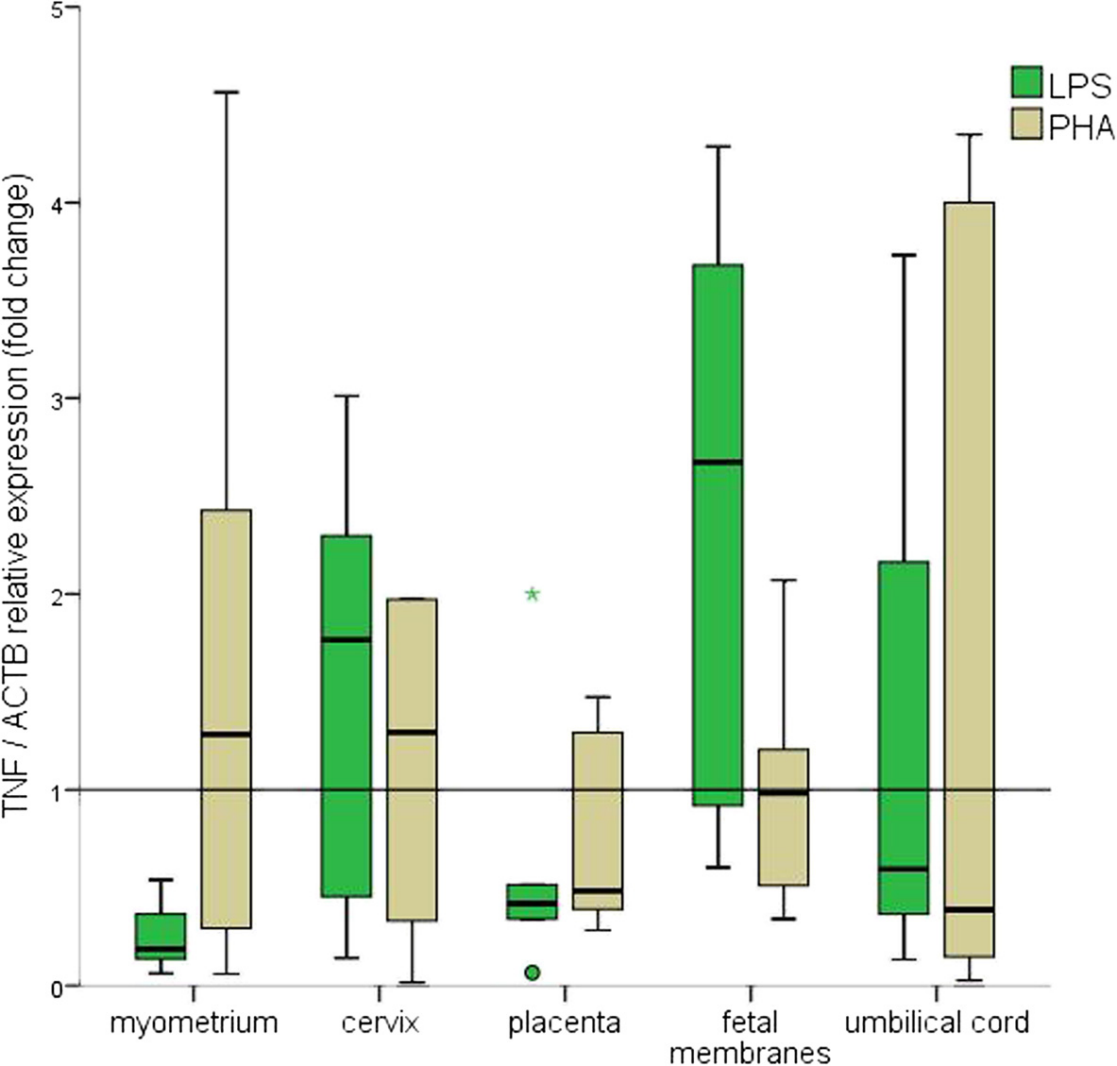
**Fold changes in *TNF *relative expression using *ACTB *as reference.** The 2^-ΔCt^ method was used to calculate fold changes. The values represent median fold change with interquartile ranges; Outlier values are represented by circles and asterisks.

**Figure 4:**
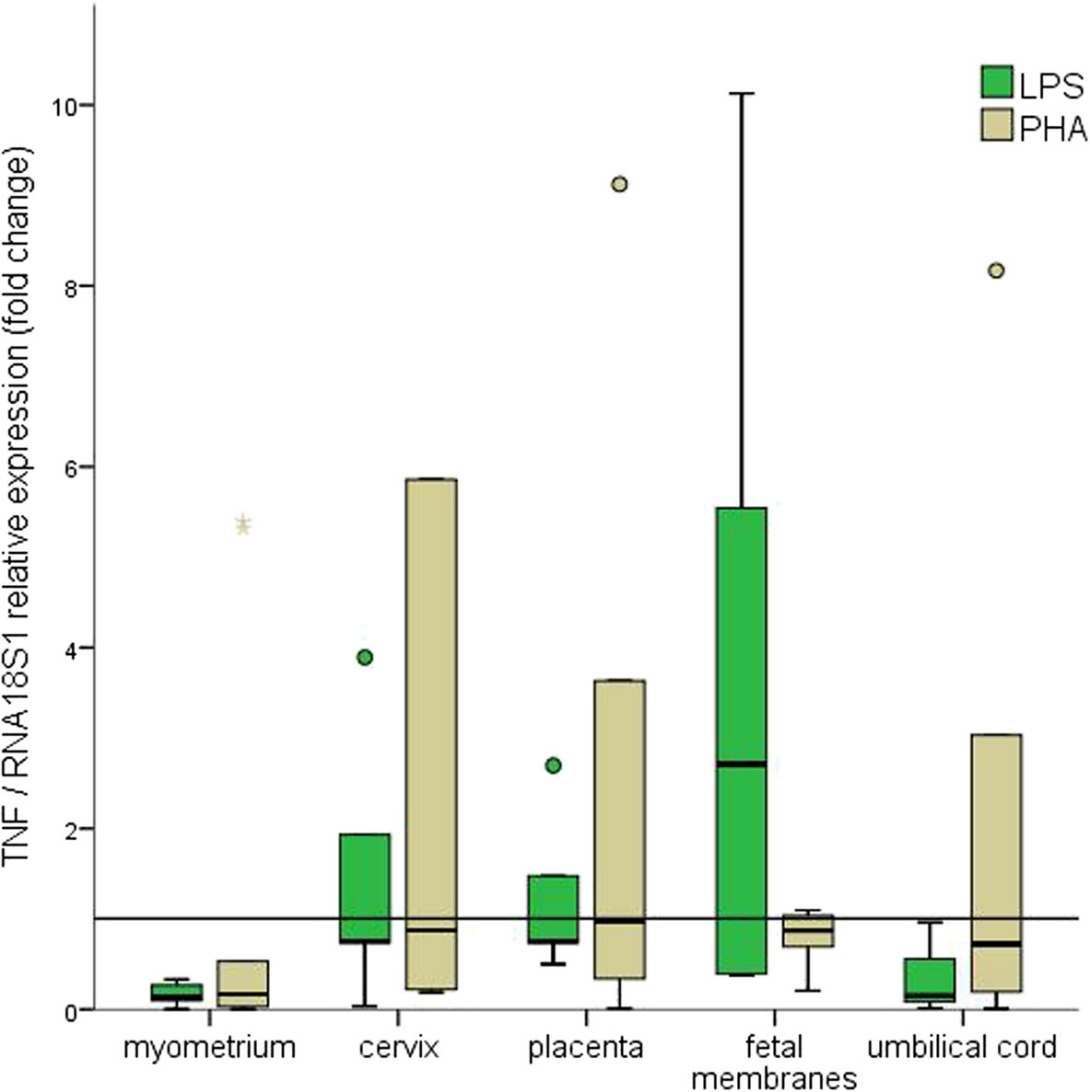
**Fold changes in *TNF *relative expression using *RNA18S1 *as reference.** The 2^-ΔCt^ method was used to calculate fold changes. The values represent median fold change with interquartile ranges; Outlier values are represented by circles and asterisks.

All tissues were responsive to the mitogen stimuli, as demonstrated by the changes in *TNF* relative expression. This behavior was consistent regardless of whether *GAPDH*, *ACTB* or *RNA18S1* were used for normalization (Figures 2, 3 and 4).

Stability measures (*M*) of housekeeping gene expression after mitogen stimulation in the various reproductive tissues are shown in Table 1. In every case, the *M* value of analyzed genes was clearly below the 1.5 threshold, meaning that genetic expression of those genes remained stable independent of experimental conditions.

**Table 1:** Stability and pairwise variation of housekeeping gene expression in reproductive tissues after mitogen stimulation

	**Stability (M)**			**Pairwise variation**
	***GAPDH***	***ACTB***	***RNA18S1***	**(V2/3)**
Umbilical cord				
(n=3 with duplicates)				
LPS	0.110*	0.095*	0.151	0.050
PHA	0.092*	0.094*	0.138	0.045
Fetal membranes				
(n=3 with duplicates)				
LPS	0.055*	0.061*	0.082	0.027
PHA	0.053*	0.050*	0.065	0.021
Placenta				
(n=3 with duplicates)				
LPS	0.105*	0.128	0.097*	0.041
PHA	0.088*	0.105	0.098*	0.032
Cervix				
(n=3 with duplicates)				
LPS	0.089*	0.102*	0.120	0.038
PHA	0.079*	0.092*	0.113	0.037
Myometrium				
(n=5 with duplicates)				
LPS	0.167*	0.196	0.192*	0.060
PHA	0.149*	0.160*	0.180	0.056

Genes with M values <1.5 are considered stable. Asterisks show the pair of genes from which pairwise variation was calculated. V2/3 <0.15 indicates that those two genes are sufficient for normalization.

Finally, the number of constitutive genes required for a reliable normalization in expression analyses was determined for each tissue. The pairwise variation analysis showed that *GAPDH* and *ACTB* are sufficient for gene expression normalization in cervix, fetal membranes and umbilical cord stimulated with LPS or PHA. These genes are also sufficient for normalization in myometrium that has been stimulated with LPS. *GAPDH* and *RNA18S1* are sufficient for normalization in placenta, regardless of the stimulus, and in myometrium stimulated with PHA (Table 1).

## Discussion

One of the main concerns in relative gene expression analyses is having a reliable normalization factor, namely- a reference gene that shows a constitutive expression (with high stability and low variability) regardless of experimental conditions. Despite the vast number of gene expression studies in human reproductive tissues, expression stability of housekeeping genes has been scarcely explored, especially in the placenta and to an even lesser extent in cervix and myometrium [8-13].

In this study, we aimed to evaluate the expression of some of the most common housekeeping genes in human reproductive tissues (myometrium, cervix, placenta, fetal membranes and umbilical cord) after being cultured and exposed to mitogen stimulation.

Mitogens such as LPS are commonly used in experimental models to emulate an intrauterine infection from Gram-negative bacteria and activate the characteristic proinflammatory response that follows. Such a bacterial-induced inflammatory response further induces the expression of several effector molecules, including cytokines, prostaglandins, defensins and matrix metalloproteinases [3,16,17].

In our experimental setup, reproductive tissues remained metabolically active throughout the 24-hour culture period. Placental and umbilical cord explants tended to decrease their metabolic activity, although not significantly, suggesting that the culture media we used may not be optimal for these particular tissues. In this regard, medium 199 seems to provide more suitable culture conditions for placental explants [18,19].

Nevertheless, changes in *TNF* relative expression confirmed that all tissues were responsive to the mitogen stimuli.

Expression analyses of housekeeping genes *GAPDH*, *ACTB* and *RNA18S1* showed great stability under our experimental conditions in all tissues. In every case, *M* values were far below the 1.5 stability threshold, proving that these three genes are suitable for the normalization of relative gene expression in human reproductive tissues under mitogen stimulation.

Our results differ somewhat from those of Meller et al. who found the most stable genes to be *TBP*, *SDHA* and *YWHAZ* in normal placental tissues, and from Cleal et al. who found that *UBC*, *TOP1* and *YWHAZ* were the most suitable housekeeping genes in normal term placentae [8,10].

Our results also differ from a recent application note by LifeTechnologies that shows *GAPDH* and *ACTB* have a high variability in placenta [13]. However, our results may not be fully comparable with those from LifeTechnologies since they inferred expression stability directly from Ct values while we used geNorm to analyze stability and pairwise variation from normalized Cp values.

By comparison, our results agree with, Murthi et al. who found that *RNA18S1*, *GAPDH* and, to a less extent, *ACTB* were stable in placentae from intrauterine growth restriction [11]. They also agree with Daud et al. regarding the stability of *GAPDH* and *ACTB* in cervical samples [9].

However, our experimental approach was different from all these studies since their analyses were performed in freshly obtained tissues while ours were done after tissue culture and stimulation. In these conditions *GAPDH* and *ACTB* showed a much greater stability than what was reported by the previous authors.

Due to the high stability of housekeeping gene expression, pairwise variation analyses showed that only two reference genes are required for adequate normalization in each experimental condition: *GAPDH* and *ACTB* being optimal in cervix, fetal membranes and umbilical cord, while *GAPDH* and *RNA18S1* are best for normalization in placenta and myometrium.

## Conclusion

Our results show that *GAPDH*, *ACTB* and *RNA18S1* are adequate references for gene expression normalization in reproductive tissues which have been stimulated with mitogens in culture.

## Competing interests

The authors declare that they have no competing interests.

## Authors’ contributions

MAH performed the experiments, analyzed data and wrote the manuscript. RVS designed the study, analyzed data and wrote the manuscript. Both authors read and approved the final manuscript.
